# Supplementary material for: Integrative in vivo analysis of the ethanolamine utilization bacterial microcompartment in Escherichia coli
Source: mSystems. 2024 Jul 18;9(8):e00750-24. doi: 10.1128/msystems.00750-24 (PMC11334477; doi:10.1128/msystems.00750-24)
Supplement: Data S2 — Details of the flux calculation through individual BMCs. [file msystems.00750-24-s0002.docx]

**Integrative *in vivo* analysis of the ethanolamine utilization bacterial microcompartment in *Escherichia coli*.**

-

Supplementary Data 2

Denis Jallet^1#^, Vanessa Soldan^2^, Ramteen Shayan^2^, Alexandre Stella^3,5^, Nour Ismail^1^, Rania Zenati^1^, Edern Cahoreau^1,4^, Odile Burlet-Schiltz^3,5^, Stéphanie Balor^2^, Pierre Millard^1,4^, Stéphanie Heux^1^.

^1^ Toulouse Biotechnology Institute, Université de Toulouse, CNRS, INRAE, INSA, Toulouse, France

^2^ Plateforme de Microscopie Electronique Intégrative, Centre de Biologie Intégrative, Université de Toulouse, CNRS, Toulouse, France

^3^ Institut de Pharmacologie et de Biologie Structurale (IPBS), Université de Toulouse, CNRS, Université Toulouse III – Paul Sabatier (UT3), Toulouse, France

^4^ MetaToul-MetaboHUB, National infrastructure of metabolomics and fluxomics, Toulouse, France

^5^ Infrastructure nationale de protéomique, ProFI, FR 2048, Toulouse, France

# Correspondance: denis.jallet@insa-toulouse.fr

Here, the flux calculations through individual BMCs based on data from literature and our experimental results are described.

**Flux through individual carboxysomes :**

**A) Based on data from Reinhold et al., 1991 (10.1139/b91-126).**

*First calculation: data from paragraph 4 on page 986*

The authors give the following V_max_ value for CO_2_ fixation:

V_max_ = 4.0 × 10^−5^ [mol/ (cm^3^ carboxysome s)]

Let the radius of a BMC be

r_BMC_ = 2 × 10^−5^ [cm]

Let the volume of a carboxysome be (NB: assuming a spherical shape for simplicity)

v_BMC_ = 4/3 x π x r^3^

= 3.35 × 10^−14^ [cm^3^]

Vmax for a single BMC will be

V_max_)_BMC_ = V_max_ x v_BMC_ [mol/(carboxysome s)]

= 1.34 × 10^−18^ [mol/(carboxysome s)] or 1.34 × 10^−3^ [fmol/(carboxysome s)]

= 4.82 × 10^−15^ [mol/( carboxysome h)] or 4.82 [fmol/( carboxysome h)]

*Second calculation: data from paragraph 2 on page 987*

The authors mention the following experimental V_max_ value for CO_2_ fixation (derived from the work by Price and Badger 1989 (10.1139/b91-124)):

V_max_._exp_)_cell_ = 2.6 × 10^−18^ [mol/ (cell s)]

= 9.36 × 10^−15^ [mol/ (cell h)]

Let’s assume there are N_BMC_)_cell_ = 6 carboxysomes per bacterium as mentioned by the authors in their text.

The V_max_ on a per carboxysome basis will be:

V_max_._exp_)_BMC_ = V_max_._exp_)_cell_ / N_BMC)cell_

= 4.33 × 10^−19^ [mol/ (carboxysome s)] or 4.33 × 10^−4^ [fmol/ (carboxysome s)]

= 1.56 × 10^−15^ [mol/ (carboxysome h)] or 1.56 [fmol/ (carboxysome h)]

**B) Based on data from Mangan and Brenner, 2014 (10.7554/eLife.02043).**

Table 3: Carboxylation rate 8.2 × 10^−8^ [picomoles/(cell s)] assuming a high HCO_3_^-^ transport rate resulting in 30 mM HCO_3_^-^ cytosolic pool.

V)_cell_ = 8.2 × 10^−8^ [picomol/(cell s)]

= 8.2 × 10^−8^ x 10^-12^ x 3.6 × 10^3^ [mol/(cell h)]

= 2.95 × 10^−16^ [mol/(cell h)] or 0.295 [fmol/(cell h)]

Let’s assume there are N_BMC_)_cell_ = 6 carboxysomes per bacterium (NB: same assumption as in Reinhold et al., 1991).

V)_BMC_ = V)_cell_ / N_BMC_)_cell_

= 4.9 × 10^−17^ [mol/(carboxysome h)] or 0.049 [fmol/(carboxysome h)]

= 1.33 × 10^−20^ [mol/(carboxysome s)] or 1.33 × 10^−5^ [fmol/(carboxysome s)]

Table 4: Carboxylation rate 5.4 × 10^−8^ [picomol/(cell s)] assuming a lower HCO_3_^-^ transport rate.

V)_cell_ = 1.94 × 10^−16^ [mol/(cell h)] or 0.194 [fmol/(cell h)]

V)_BMC_ = 3.2 × 10^−17^ [mol/(carboxysome h)] or 0.032 [fmol/(carboxysome h)]

**Flux through individual Pdu BMCs :**

**A) Work by Jakobson et al., 2017 (10.1371/journal.pcbi.1005525)**

Assuming that PduP/Q limit 1,2-PD assimilation within the *S*. *enterica* Pdu BMCs *in vivo*, the authors calculated the following 1,2-PD conversion flux per cell:

F_1,2-PD_)_cell_ = 2.97 × 10^−13^ [µmol/(cell s)]

= 1.07 × 10^−9^ [µmol/(cell h)]

= 1.07 × 10^−15^ [mol/(cell h)] or 1.07 [fmol/(cell h)]

Let assume the number of Pdu BMCs per cell to be (based on Kennedy et al., 2022; 10.1128/jb.00576-21)

N_BMCs_)_cell_ = 3

The 1,2-PD conversion flux per Pdu BMC will then be

V_1,2-PD_)_BMC_ = F_cell_ / N_BMCs_

= 1.07 / 3 = 0.356 [fmol/(BMC h)]

**B) Work by Jakobson et al., 2018 (10.1038/s41598-018-26399-0)**

For 1,2-PD assimilation, pathway flux of roughly

F_1,2-PD_)_cell_ = 2.97 × 10^−13^ [µmol/(cell s)] (based on observations by Sampson and Bobik, 2008: 10.1128/JB.01925-07)

The results will be identical to that above.

**Flux through individual Eut BMCs :**

Based on our experimental data, let the EA uptake rate be

q_S_)_EA_ = 7 [mmol.g_DW_^-1^.h^-1^]

Let the number of Eut BMCs per cell be

N_BMCs_)_cell_  = 6

Based on literature, let the dry weight of an *E*. *coli* cell be

m_cell_ = 258 [fg] (BioNumbers assuming a doubling rate of 60 min)

= 2.58 × 10^−13^ [g]

The number of *E*. *coli* cells in 1 g DW will be

N_cells_ = 1 / m_cell_

= 1 / (2.58 × 10^−13^) = 3.88 × 10^12^ cells

The number of Eut BMCs in 1 g DW will be

N_BMCs_ = N_cells_ x N_BMCs_)_cell_

= 3.88 × 10^12^ x 6 = 2.33 × 10^13^

The EA conversion flux per BMC will be

V_EA_)_BMC_  = q_S_)_EA_ / N_BMCs_

= 7 / (2.33 × 10^13^) = 3.00 × 10^−13^ [mmol/(BMC h)]

= 3.00 × 10^−16^ [mol/(BMC h)]

= 0.300 [fmol/(BMC h)]

**Glycolytic flux based on our data:**

Based on our modelling data, let the glycolytic flux be:

F_glyco_ = 2.5 [mmol.g_DW_^-1^.h^-1^]

Based on literature, let the dry weight of an *E*. *coli* cell be

m_cell_ = 258 [fg] (BioNumbers assuming a doubling rate of 60 min)

= 2.58 × 10^−13^ [g]

The number of *E*. *coli* cells in 1 g DW will be

N_cells_ = 1 / m_cell_

= 1 / (2.58 × 10^−13^) = 3.88 × 10^12^ cells

The glycolytic flux per cell will be:

F_glyco)cell_ = F_glyco_ / N_cells_

= 2.5 / (3.88 × 10^12^) [mmol/(cell h)]

= 7.2 × 10^-13^ [mmol/(cell h)]

= 7.2 × 10^-16^ [mol/(cell h)]

= 0.72 [fmol/(cell h)]

PEP step)

Then F_glyco)cell_ = 2.016 [fmol/(cell h)]
