# Supplementary material for: Integrative in vivo analysis of the ethanolamine utilization bacterial microcompartment in Escherichia coli
Source: mSystems. 2024 Jul 18;9(8):e00750-24. doi: 10.1128/msystems.00750-24 (PMC11334477; doi:10.1128/msystems.00750-24)
Supplement: Supplemental figures — Fig. S1-S9. [file msystems.00750-24-s0003.pdf]

Supplementary figures

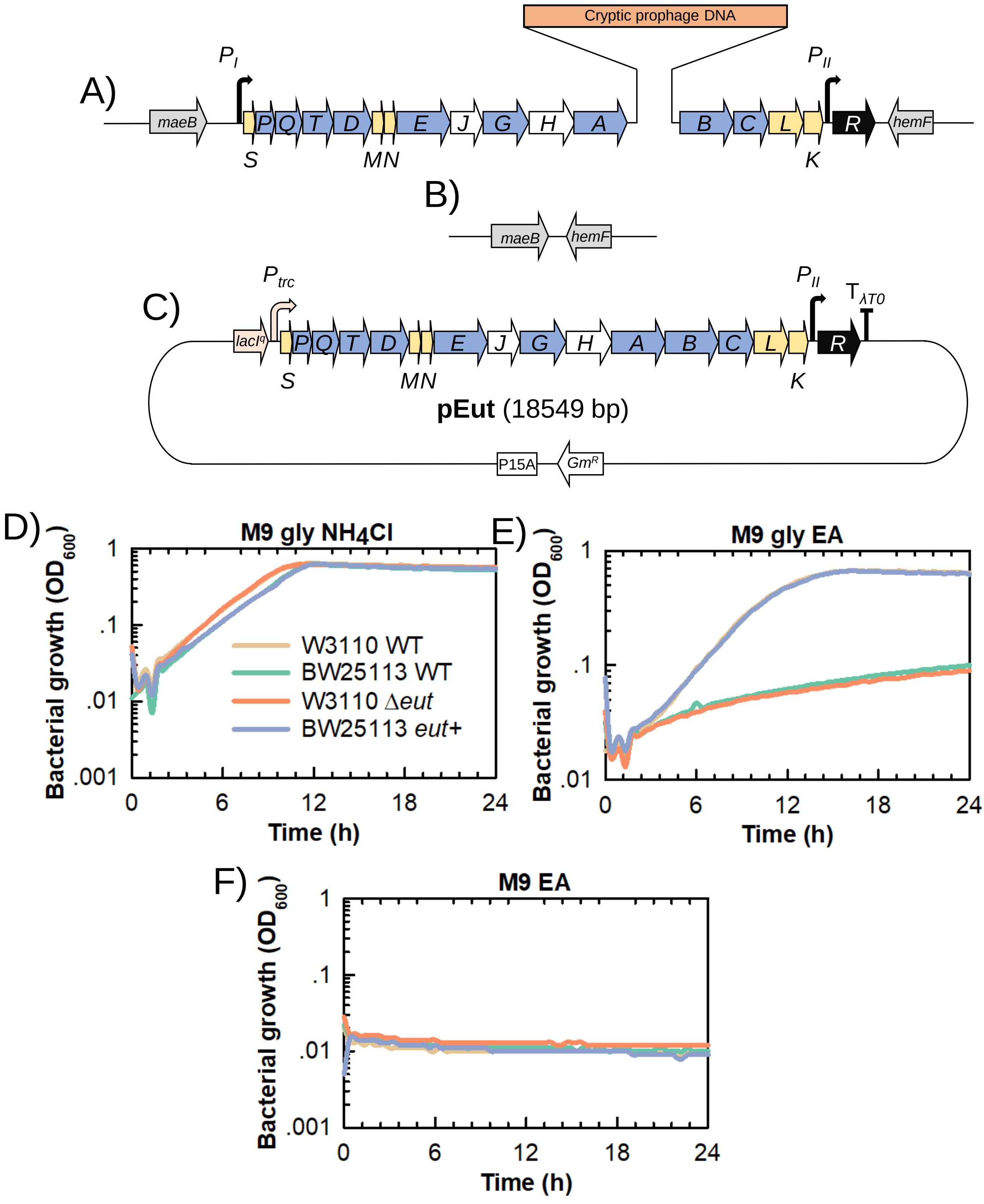

Figure S1. Capacity to utilize EA as a N source in *Escherichia coli* K-12 and mutant derivatives. Schematic representation of the *eut* operon in A) the chromosome of BW25113 WT where it harbors a 6,9 kb prophage DNA insertion downstream *eutA*, B) the chromosome of W3110 *Δeut* where the entire operon was scarlessly removed by genome editing and C) the pEut plasmid where the *eut* operon from W3110 WT was cloned into pSEVA661 under control of the IPTG-inducible P_{trc} promoter. The BW25113 *eut+* mutant was generated by scarlessly removing the prophage DNA insertion through genome editing, thus reconstituting an uninterrupted *eut* operon (yielding the same *eut* operon as in W3110 WT). D) to F) Plate reader measurement of optical density at 600 nm over time. The strains were grown in D) M9 derivative with glycerol as the C source and NH_4Cl as the N source; E) M9 derivative with glycerol as the C source and EA as the sole N source, including also vitamin B12; F) M9 derivative with EA (160 mM) as the C and N source, including also 200 nM vitamin B12. Data shows a representative experiment out of at least n = 3 independent replicates.

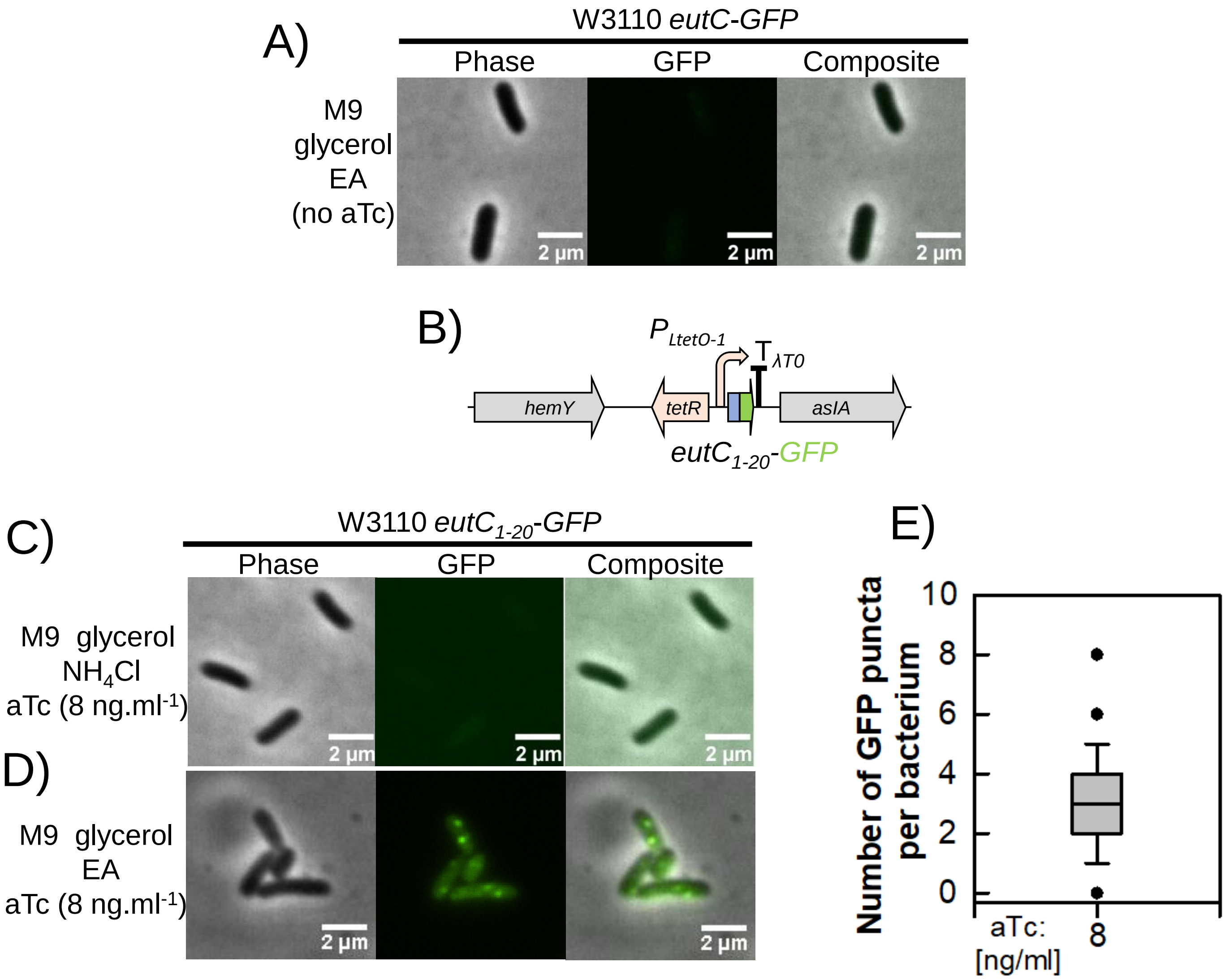

Figure S2. Epifluorescence microscopy to determine the EutC-GFP and EutC$_{1\text{-}20}$-GFP subcellular localization. A) Representative micrograph showing a phase contrast image, GFP fluorescence signal as well as a composite image from W3110 *eutC-GFP* grown in M9 glycerol EA vit B12 without any aTc added. B) Schematic representation of the P$_{LtetO\text{-}1}$::eutC$_{1\text{-}20}$-GFP expression cassette. This construct was inserted at the SS9 safe locus site in W3110 WT to yield W3110 *eutC$_{1\text{-}20}$-GFP*. C) and D) Representative micrographs showing phase contrast images, GFP fluorescence signal as well as composite images from W3110 *eutC$_{1\text{-}20}$-GFP* grown in C) M9 glycerol NH_4Cl with 8 ng.ml^{-1} aTc and D) M9 glycerol EA vit B12 with 8 ng.ml^{-1} aTc. E) Box plot indicating the number of GFP puncta per bacterium out of n = 30 individual cells for cultures grown in M9 glycerol EA vit B12 with 8 ng.ml^{-1} aTc.

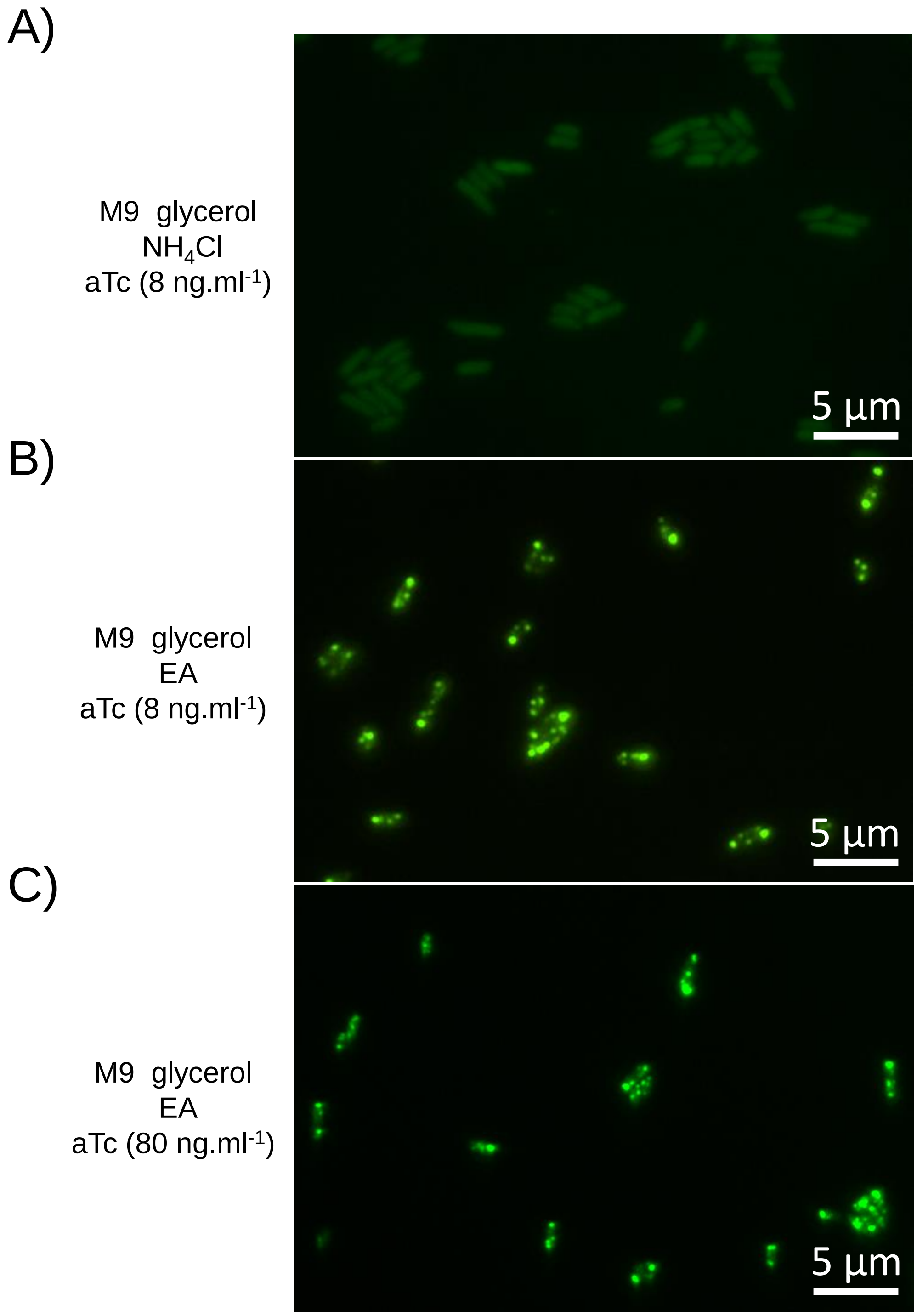

Figure S3. Wider views of the GFP channel for W3110 eutC-GFP grown in various media. Micrographs showing the strain grown in A) M9 glycerol NH_4Cl with 8 $ng.ml^{-1}$ aTc, B) M9 glycerol EA vitamin B12 with 8 $ng.ml^{-1}$ aTc and C) M9 glycerol EA vitamin B12 with 80 $ng.ml^{-1}$ aTc. Scale bars indicate 5 µm.

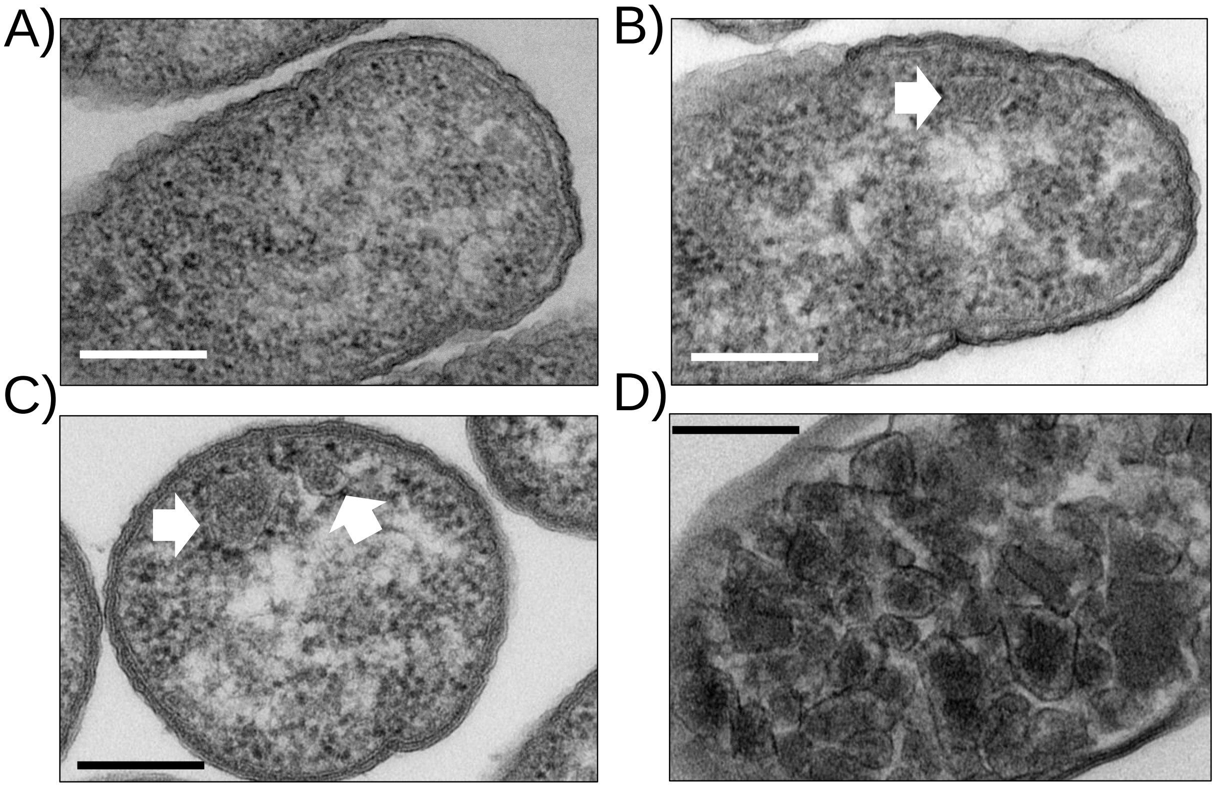

Figure S4. Observation of the *Escherichia coli* K-12 W3110 Eut BMCs by TEM. A) W3110 WT in M9 glycerol NH_4Cl; B) W3110 WT in M9 glycerol EA vit B12; C) W3110 WT in LB EA vit B12 and D) W3110 Δeut pEut_WT in LB 20 µM IPTG. The filled white arrows indicate Eut BMCs in B and C. Scale bars show 200 nm.

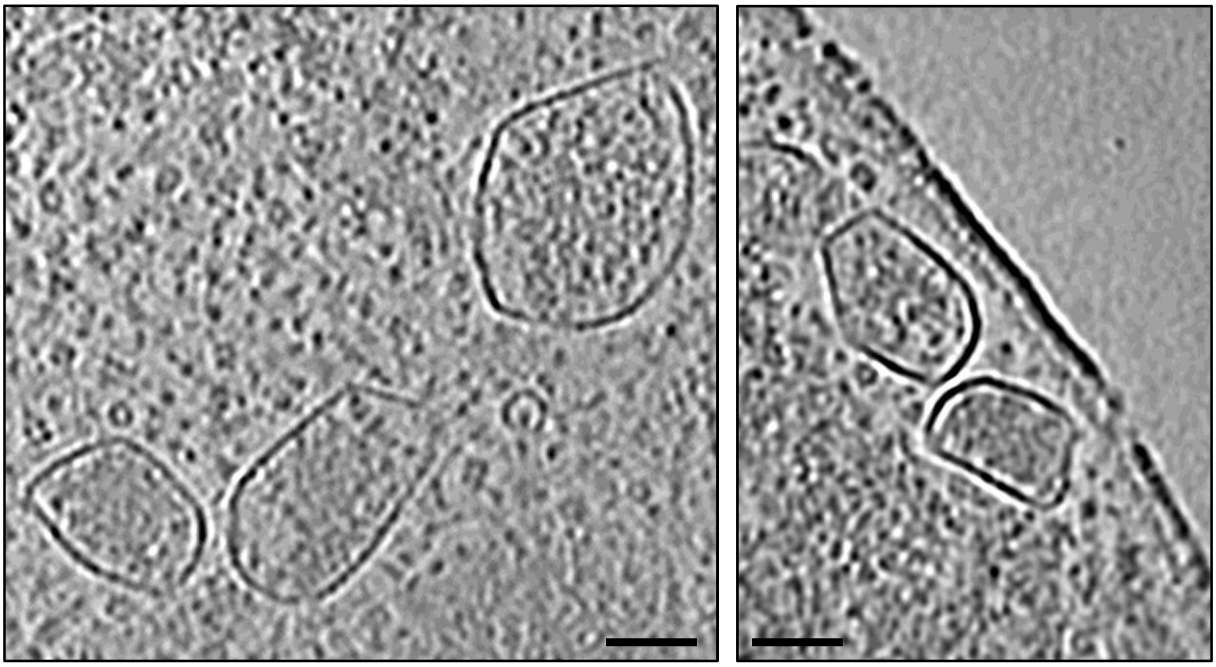

Figure S5. CryoET observation of the Eut BMCs formed by W3110 Δ*eut* pEut. The *eut* overexpressor strain was grown in M9 glycerol EA with 20 µM IPTG. Representative electron cryotomogram slices are shown. The scale bars indicate 50 nm.

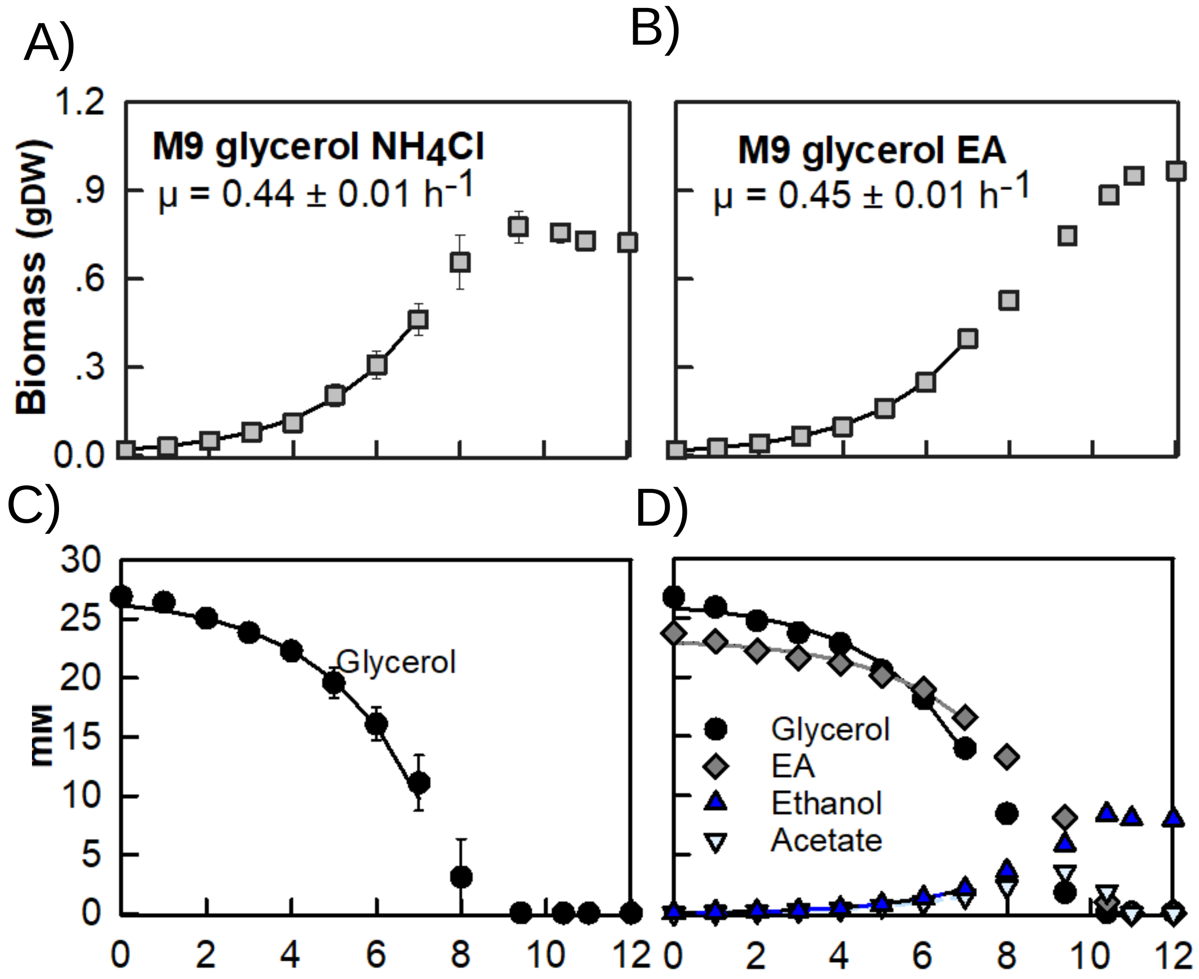

Figure S6. Physiological characterization of *Escherichia coli* K-12 W3110 WT grown in M9 glycerol containing NH_4Cl or EA as the sole N source. Cultures were grown aerobically in A) and C) M9 glycerol (30 mM) NH_4Cl (25 mM) or B) and D) M9 glycerol (30 mM) EA (25 mM) vit B12 (200 nM). A) and B) Biomass accumulation (grams dry weight, gDW); C) and D) Exometabolome profiling by ^{1}H-NMR. Data is the average of n = 3 biological replicates, bars show SD. The solid lines are the best fits obtained with PhysioFit.

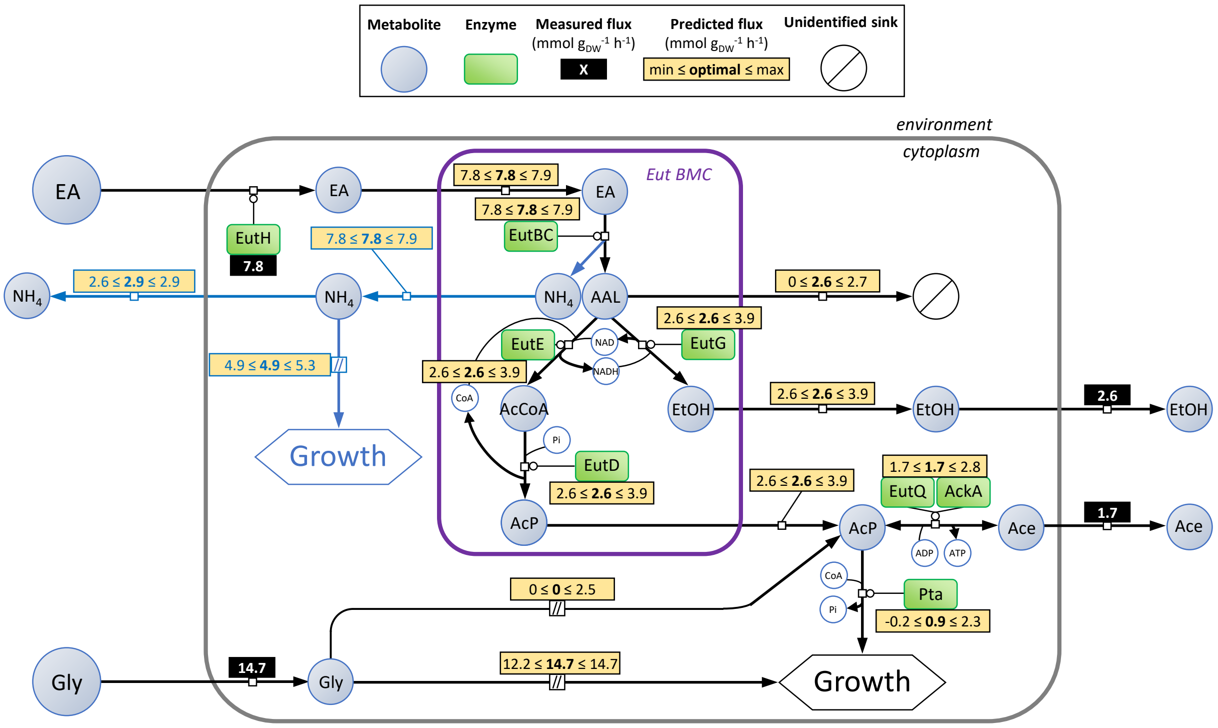

Figure S7. FBA/FVA analysis of EA metabolism in *E. coli* K-12 W3110. Black rectangles indicate the experimentally determined exchange fluxes for W3110 WT grown in M9 glycerol (30 mM) EA (25 mM) (data from Fig.S5). Yellow rectangles report the predicted fluxes. The BMC-associated fluxes were deduced assuming that NADH and CoA-SH are recycled internally while ATP is recycled within the cytosol. We also assumed that acetate and ethanol were produced from the BMCs. The non-constrained fluxes were simulated by flux balance analysis (optimal value: bold font) combined with flux variability analysis (to determine possible value ranges while maintaining 99% of the fitness). Our model aimed at maximizing ATP production while the growth rate was set to 0.45 h^{-1} to match the experimental data. Flux values are given in $mmol.g_{DW}^{-1}.h^{-1}$. AAL: acetaldehyde; AcCoA: acetyl-CoA; AcP: acetyl-phosphate; EtOH: ethanol; Gly: glycerol; NH_4: ammonium.

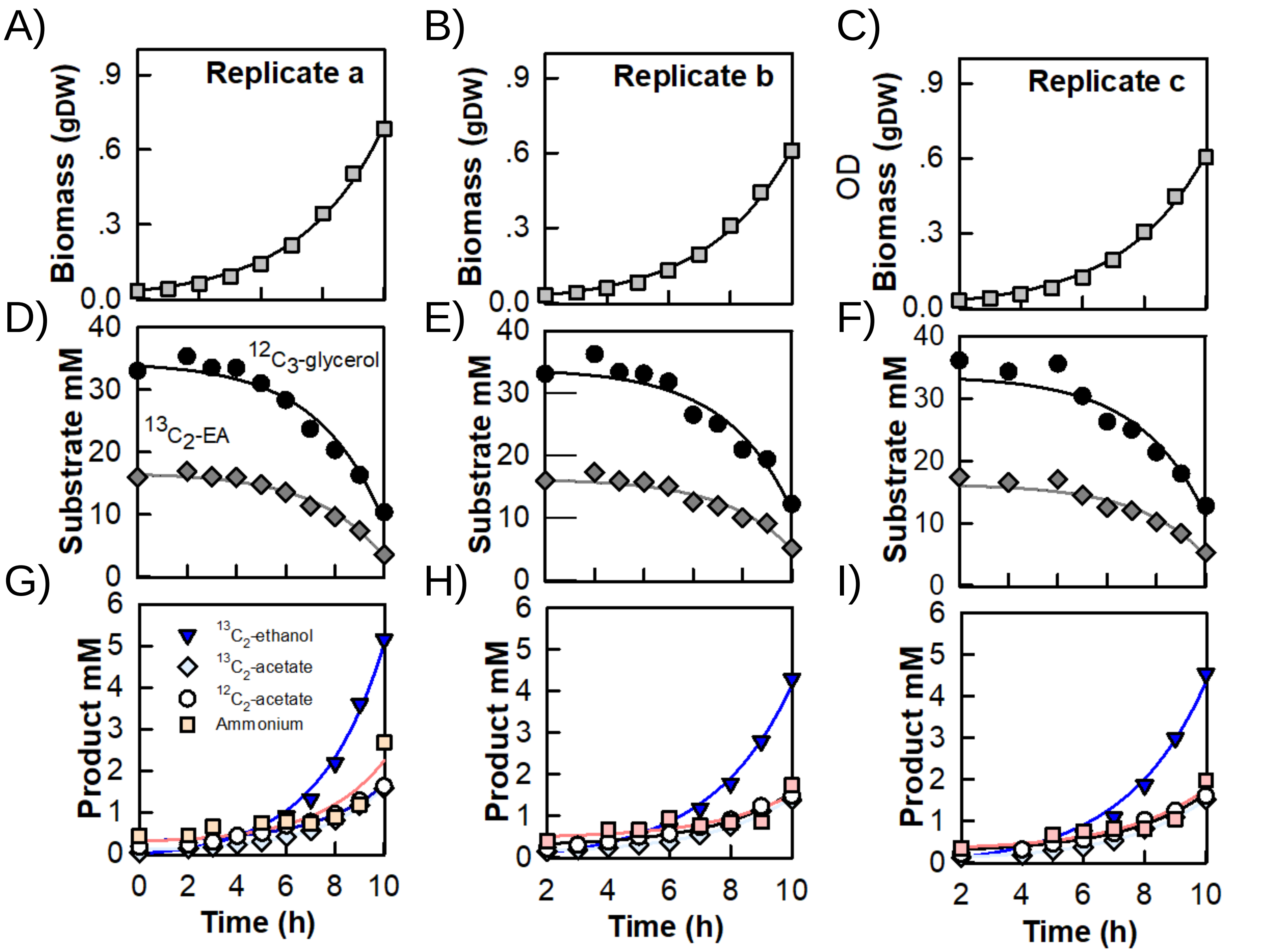

Figure S8. The dynamic isotopic model fits the experimental data satisfactorily. W3110 WT cultures (3 biological replicates named a, b and c) were grown aerobically in M9 medium containing $^{12}C_3$-glycerol and $^{13}C_2$-EA. A), B) and C) Biomass accumulation (grams dry weight, gDW) in biological replicates a, b and c. Exometabolome composition was determined by ^{1}H-NMR. D), E) and F) $^{12}C_3$-glycerol and $^{13}C_2$-EA uptake in biological replicates a, b and c. G), H) and I) $^{13}C_2$-EtOH, $^{13}C_2$-acetate, $^{12}C_2$-acetate and ammonium production in biological replicates a, b and c. Solid lines show the best fits with our dynamic isotopic model. The experimental data from all 3 replicates were averaged to produce Figure 5.

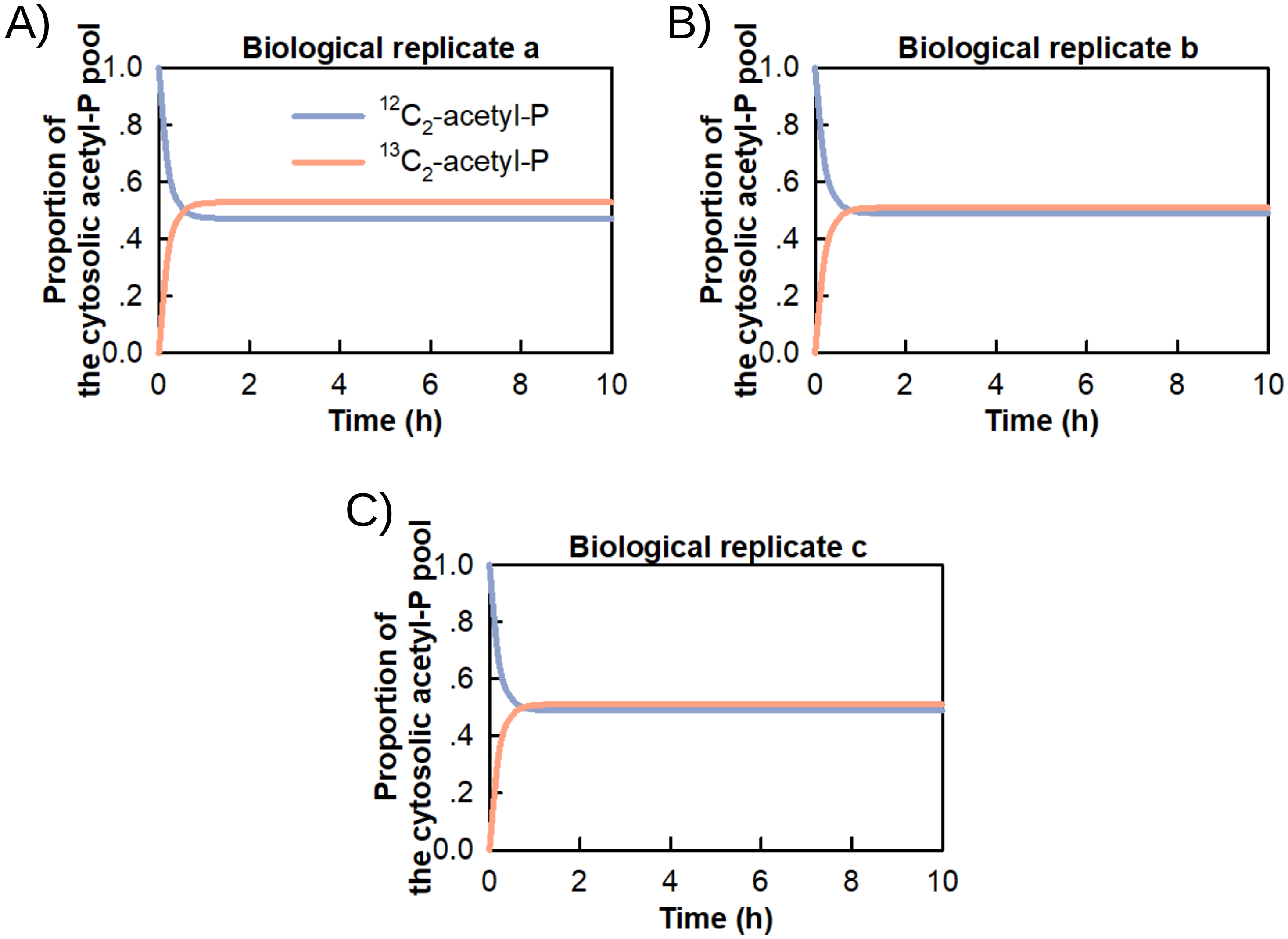

Figure S9. Predicted evolution of the cytosolic acetyl-P pool isotopic composition. The isotopic model was used to fit data from Fig.5. The predicted proportion of cytosolic $^{12}C_2$-acetyl-P (derived from glycerol) VS $^{13}C_2$-acetatyl-P (derived from EA) over time is shown for each biological replicate (A to C).

References

1. Xu, W., Dunn, C. A., O'handley, S. F., Smith, D. L. & Bessman, M. J. Three new Nudix hydrolases from Escherichia coli. J. Biol. Chem. 281, 22794–22798 (2006).
2. Rahman, M. & Shimizu, K. Altered acetate metabolism and biomass production in several Escherichia coli mutants lacking rpoS-dependent metabolic pathway genes. Mol. Biosyst. 4, 160–169 (2008).
3. Weber, H., Polen, T., Heuveling, J., Wendisch, V. F. & Hengge, R. Genome-wide analysis of the general stress response network in Escherichia coli: σS-dependent genes, promoters, and sigma factor selectivity. J. Bacteriol. 187, 1591–1603 (2005).
4. Bologna, F. P., Andreo, C. S. & Drincovich, M. F. Escherichia coli malic enzymes: two isoforms with substantial differences in kinetic properties, metabolic regulation, and structure. J. Bacteriol. 189, 5937–5946 (2007).
5. Mancini, S. & Imlay, J. A. The induction of two biosynthetic enzymes helps E scherichia coli sustain heme synthesis and activate catalase during hydrogen peroxide stress. Mol. Microbiol. 96, 744–763 (2015).
6. Heidrich, C. et al. Involvement of N-acetylmuramyl-L-alanine amidases in cell separation and antibiotic-induced autolysis of Escherichia coli. Mol. Microbiol. 41, 167–178 (2001).
7. Christensen, D. G. et al. Identification of novel protein lysine acetyltransferases in Escherichia coli. MBio 9, 10–1128 (2018).
8. Rezuchova, B., Miticka, H., Homerova, D., Roberts, M. & Kormanec, J. New members of the Escherichia coli σE regulon identified by a two-plasmid system. FEMS Microbiol. Lett. 225, 1–7 (2003).
9. Dailey, H. A. et al. The Escherichia coli protein YfeX functions as a porphyrinogen oxidase, not a heme dechelatase. MBio 2, 10–1128 (2011).
10. Vega, D. & Ayala, J. A. The DD-carboxypeptidase activity encoded by pbp4B is not essential for the cell growth of Escherichia coli. Arch. Microbiol. 185, 23–27 (2006).
11. Jaeger, T. & Mayer, C. The transcriptional factors MurR and catabolite activator protein regulate N-acetylmuramic acid catabolism in Escherichia coli. J. Bacteriol. 190, 6598–6608 (2008).
