# Supplementary material for: Integrative in vivo analysis of the ethanolamine utilization bacterial microcompartment in Escherichia coli
Source: mSystems. 2024 Jul 18;9(8):e00750-24. doi: 10.1128/msystems.00750-24 (PMC11334477; doi:10.1128/msystems.00750-24)
Supplement: Legend — for Movie S1. [file msystems.00750-24-s0004.docx]

**Supplementary Movie 1.** Visualization of the moving Eut BMCs *in vivo* in *E*. *coli* K-12 W3110. W3110 *eutC-GFP* was grown in M9 glycerol EA vitamin B12 with 8 ng.ml^-1^ aTc. Upon reaching stationary phase, samples were collected and deposited onto M9 medium agarose pads. The GFP fluorescence signal was recorded every 5 seconds for 30 seconds. The movie was assembled at a rate of 7 frames per second (i.e. acceleration 35 times).
