# Supplementary material for: Integrative in vivo analysis of the ethanolamine utilization bacterial microcompartment in Escherichia coli
Source: mSystems. 2024 Jul 18;9(8):e00750-24. doi: 10.1128/msystems.00750-24 (PMC11334477; doi:10.1128/msystems.00750-24)
Supplement: Supplemental tables — Tables S1 to S5. [file msystems.00750-24-s0005.docx]

| **Primer name** | **Sequence** |
| --- | --- |
| F_bb1 | TGAGCTAGCTGTCAAGGATCC |
| R_bb1 | AAGTTAAAATAAGGCTAGTCCGTTAT |
| F_eutGgRNA | ATCCTTGACAGCTAGCTCAGTCCTAGGTATAATACTAGTCGGCACACCTTCGGTCAATG |
| R_eutGgRNA | ACTAGCCTTATTTTAACTTGCTATTTCTAGCTCTAAAACCATTGACCGAAGGTGTGCCG |
| F_bb2 | CATAGTTAAGCCAGCCCCGA |
| R_bb2 | TGTCGTGCCAGCTGCATTAAT |
| F_UA1 | TTAATGCAGCTGGCACGACAGCGGCCGCAGCTGGCGGAGATCACCTTG |
| R_UA1 | ACTCCCGCATCTTACCCTGAATATTCAGGGTAAGC |
| F_LA1 | TCAGGGTAAGATGCGGGAGTGGGGGTGA |
| R_LA1 | TCGGGGCTGGCTTAACTATGGCGGCCGCATTCACTGGCATCGCACCG |
| F_HD1 | CATAGTTAAGCCAGCCCCGA |
| R_HD1 | TGTCGTGCCAGCTGCATTAAT |
| F_SS9gRNA | ATCCTTGACAGCTAGCTCAGTCCTAGGTATAATACTAGTTCTGGCGCAGTTGATATGTA |
| R_SS9gRNA | ACTAGCCTTATTTTAACTTGCTATTTCTAGCTCTAAAACTACATATCAACTGCGCCAGA |
| F_UA2 | TTAATGCAGCTGGCACGACAGAATTCCCGACGTCCATCCAGCCC |
| F_UA2 | GATATAATAAACCTGTTTGACATATCAACTGCGCCAGAGG |
| F_LA2 | TCAAACAGGTTTATTATATCGCGTTGATTATTGATGC |
| R_LA2 | TCGGGGCTGGCTTAACTATGGAATTCATAACCCGCCACAGTAGTTC |
| F_bb3 | TACCAGTGGCGGCTAGTCTTGGACTCCTGTTG |
| R_bb3 | TCGCCGCCTGTGCAGGATTAGCAGATGTGTG |
| F_eut1 | TCTGCTAATCCTGCACAGGCGGCGACTCATG |
| R_eut1 | GCGTAATCGCCCGCTGGGAGACTTTTTTCGCC |
| F_eut2 | AAAGTCTCCCAGCGGGCGATTACGCTGCTCAAC |
| R_eut2 | ATGCGCGCCCGCGCCAATCACCGTGGCGCGC |
| F_eut3 | CACGGTGATTGGCGCGGGCGCGCATACCCT |
| R_eut3 | AGTCCAAGACTAGCCGCCACTGGTACGCTGGG |

**Supplementary Table 1**. Primers used in the present study.

| **Position** | **Protein** | **Gene**  **orientation** | **UniProt Id** | **Annotated Pfam domains** | **Available information** | **Present in *S*. *enterica* LT2?** | **% Identity** | **% Similarity** |
| --- | --- | --- | --- | --- | --- | --- | --- | --- |
| Upstream  *eut* | NudK | Plus | P37128 | PF00293, NUDIX domain | GDP-mannose hydrolase^1^. May play a role in biofilm formation or growth onto solid substrates. | Yes | 88 | 95 |
|  | YpfG | Plus | P76559 | PF06674, Protein of unknown function (DUF1176) | Unknown. | Yes, but followed by a gene  insertion in the *ypfG*-*tktB* intergenic region encoding a putative cytoplasmic protein (Uniprot: Q7CQ26) | 73 | 82 |
|  | TktB | Minus | P33570 | PF00456, Transketolase thiamine diphosphate binding domain PF02779, Transketolase pyrimidine binding domain PF02780, Transketolase C-terminal domain | Participates in the non-reductive branch of the Pentose Phosphate Pathway^2^. Expression positively regulated by the σ^S^ stress-response sigma factor^3^. | Yes | 93 | 96 |
|  | TalA | Minus | P0A867 | PF00923, Transaldolase/Fructose-6-phosphate aldolase | Participates in the non-reductive branch of the Pentose Phosphate Pathway^2^. Expression positively regulated by the σ^S^ stress-response sigma factor^3^. | Yes | 90 | 94 |
|  | MaeB | Plus | P76558 | PF00390, Malic enzyme N-terminal domain PF03949, Malic enzyme NAD binding domain PF01515, Phosphate acetyl/butaryl transferase | NADP dependent malic enzyme^4^. Catalyzes the reductive decarboxylation of malate into pyruvate. Ensures a link between the TCA cycle and lower glycolysis. | Yes, but followed by a gene insertion in the *maeB-eutS* intergenic region encoding an IS200 family transposase | 97 | 94 |
| *eut* operon | EutS | Plus | P63746 | PF00936, BMC domain | Hexameric BMC shell protein. | Yes | 95 | 99 |
|  | EutP | Plus | P0A208 | PF10662, RAS-like GTPase superfamily domain | Probable acetate kinase.  May play a role in positioning the Eut BMCs. | Yes | 84 | 91 |
|  | EutQ | Plus | P76555 | PF06249, EutQ | Probable acetate kinase. | Yes | 88 | 93 |
|  | EutT | Plus | P65643 | PF01923, Cobalamin adenosyltransferase | Converts cyanocobalamin to adenosylcobalamin. | Yes | 88 | 93 |
|  | EutD | Plus | P77218 | PF01515, Phosphate acetyl/butaryl transferase | Phosphate acetyltransferase | Yes | 89 | 95 |
|  | EutM | Plus | P41791 | PF00936, BMC domain | Hexameric BMC shell protein. | Yes | 96 | 97 |
|  | EutN | Plus | P0AEJ8 | PF03319, EutN/carboxysome | Pentameric BMC shell protein. | Yes | 88 | 93 |
|  | EutE | Plus | P77445 | PF00171, Aldehyde dehydrogenase family | Acetaldehyde dehydrogenase. | Yes | 94 | 97 |
|  | EutJ | Plus | P77277 | PF14450, Cell division protein FtsA | Chaperone of unknown function. | Yes | 89 | 94 |
|  | EutG | Plus | P76553 | PF00465, Iron-containing alcohol dehydrogenase | Alcohol dehydrogenase. | Yes | 81 | 86 |
|  | EutH | Plus | P76552 | PF04346, EutH | Ethanolamine transporter. | Yes | 95 | 96 |
|  | EutA | Plus | P76551 | PF06277, EutA | Ethanolamine ammonia-lyase reactivase. | Yes | 90 | 95 |
|  | EutB | Plus | P0AEJ6 | PF06751, EutB | Ethanolamine-ammonia lyase large subunit | Yes | 98 | 99 |
|  | EutC | Plus | P19636 | PF05985, EutC | Ethanolamine-ammonia lyase small subunit | Yes | 91 | 94 |
|  | EutL | Plus | P76541 | PF00936, BMC domain | Trimeric BMC shell protein | Yes | 94 | 98 |
|  | EutK | Plus | P76540 | PF00936, BMC domain PF16365, EutK C-terminus | BMC shell protein with a potential DNA  binding motif | Yes | 78 | 83 |
|  | EutR | Plus | P36547 | PF12833, Helix-turn-helix domain | DNA-binding transcriptional activator | Yes, but followed by 2 gene  insertions in the *eutR*-*hemF* intergenic region (Uniprot: Q9ZFU5 and Q9ZFU6) | 92 | 97 |
| Downstream  *eut* | HemF | Minus | P36553 | PF01218, Coproporphyrinogen III oxidase | Replaces HemN in the heme biosynthetic pathway when peroxide is present^5^. Only functions under aerobic conditions. | Yes | 92 | 96 |
|  | AmiA | Minus | P36548 | PF01520, N-acetylmuramoyl-L-alanine amidase | Plays a role in cell wall peptidoglycan recyling^6^. Involved in the septation process during cell division. | Yes | 88 | 95 |
|  | YpeA | Plus | P76539 | PF00583, Acetyltransferase (GNAT) family | Unknown. Does not seem to act as a lysine acetyl transferase^7^. | Yes | 94 | 98 |
|  | YfeZ | Plus | P76538 | PF11143, Protein of unknown function (DUF2919) | Unknown. Putative inner membrane protein. | Yes | 74 | 81 |
|  | YfeY | Plus | P76537 | PF06572, Protein of unknown function (DUF1131) | Unknown. Expression regulated by the σ^E^ envelope stress-response sigma factor^8^. | Yes | 82 | 91 |
|  | YfeX | Plus | P76536 | PF04261, Dyp-type peroxidase N-terminal | Converts protoporphyrinogen IX and coproporphyrinogen III into porphyrins^9^. Expressed under anaerobic conditions. May be required for the synthesis of some components of the anaerobic respiratory chain. | Yes | 93 | 96 |
|  | YfeW | Minus | P77619 | PF00144, Beta-lactamase | Penicillin binding protein PBP4B^10^. Unknown function. | Yes, but not at the same  genomic location (in the intergenic region upstream *nudK*) | 73 | 84 |
|  | MurP | Minus | P77272 | PF00367, Phosphotransferase system EIIB PF02378, Phosphotransferase system EIIC | N-acetylmuramic acid/anhydro-N-acetylmuramic acid transporter. Plays a role in cell wall peptidoglycan recycling^11^. | No | ND | ND |

**Supplementary Table 2.** Proteins encoded within the EUT1 locus of *E*. *coli* K-12 W3110. Proteins encoded within the *eut* operon are highlighted in orange. Sequence alignments were performed using BlastP to determine the percentages of primary structure identity and similarity with the EUT1 locus proteins of *Salmonella enterica* subsp. *enterica* serovar Typhimurium LT2.

| Protein | Fold-change | p-value | Predicted function |
| --- | --- | --- | --- |
| eutP | 368,9 | 1,22E-09 | Probable acetate kinase |
| eutM | 325,1 | 8,87E-08 | Hexameric BMC shell protein |
| eutT | 206,6 | 6,23E-09 | Corrinoid adenosyltransferase |
| eutE | 160,9 | 1,41E-07 | Acetaldehyde dehydrogenase |
| amtB | 150,6 | 3,42E-07 | Ammonium transporter, AMT family |
| eutS | 148,6 | 1,35E-07 | Hexameric BMC shell protein |
| eutQ | 134,1 | 9,01E-09 | Probable acetate kinase |
| eutB | 110,5 | 3,13E-09 | Ethanolamine-ammonia lyase large subunit |
| eutG | 84,8 | 1,18E-08 | Alcohol dehydrogenase |
| glnK | 73,5 | 1,47E-06 | Nitrogen assimilation regulatory protein for GlnL, GlnE and AmtB |
| eutC | 66,1 | 8,99E-09 | Ethanolamine-ammonia lyase small subunit |
| eutH | 65,7 | 4,73E-08 | Ethanolamine permease |
| eutD | 58,9 | 7,28E-08 | Phosphate acetyltransferase |
| eutL | 48,3 | 9,27E-08 | Trimeric BMC shell protein |
| eutR | 29,8 | 3,70E-05 | DNA-binding transcriptional activator |
| eutK | 22,6 | 3,69E-08 | BMC shell protein |
| eutA | 19,6 | 1,22E-07 | Ethanolamine ammonia-lyase reactivase |
| eutN | 15,5 | 5,74E-07 | Pentameric BMC shell protein |
| priA | 7,1 | 1,80E-06 | Primosomal protein n' (replication factor y) |
| ddpX | 7,0 | 3,74E-03 | D-Ala-D-Ala dipeptidase, Zn-dependent |
| acrB | 6,8 | 4,01E-07 | Multidrug efflux pumpRND permease |
| hrpA | 6,1 | 9,99E-06 | ATP-dependent RNA helicase |
| ddpA | 6,0 | 1,79E-06 | Putative d,d-dipeptide abc transporter periplasmic binding protein |
| eutJ | 5,4 | 8,05E-08 | Putative chaperone |
| oppA | 5,1 | 7,28E-05 | Oligopeptide abc transporter periplasmic binding protein |
| uxuA | 4,3 | 1,06E-06 | D-mannonate dehydratase |
| guaD | 4,1 | 1,40E-06 | Guanine deaminase |
| ampC | 3,9 | 3,17E-06 | β-lactamase |
| cbl | 3,7 | 3,81E-04 | DNA-binding transcriptional activator Cbl |
| ybiO | 3,7 | 1,18E-04 | Mechanosensitive channel |
| gcvP | 3,5 | 4,33E-10 | Glycine decarboxylase |
| glnL | 2,9 | 5,38E-06 | Two-component system, ntrc family |
| yqjH | 2,9 | 1,80E-02 | NADPH-dependent ferric chelate reductase |
| cycA | 2,8 | 9,46E-06 | D-serine/alanine/glycine/:H^+^symporter |
| rsmA | 2,7 | 1,21E-07 | rRNA dimethyltransferase |
| astD | 2,6 | 1,35E-07 | Succinylglutamate-semialdehyde dehydrogenase |
| glnA | 2,6 | 2,13E-06 | Glutamine synthetase |
| astC | 2,6 | 1,53E-05 | Succinylornithine transaminase |
| glnG | 2,6 | 4,28E-06 | Two-component system, ntrc family |
| rnpA | 2,4 | 4,14E-06 | Ribonuclease P protein |
| asnB | 2,4 | 1,85E-06 | Asparagine synthase (glutamine-hydrolysing) |
| hisQ | 2,3 | 1,65E-03 | Lysine/arginine/ornithine ABC transporter, membrane subunit |
| gcvH | 2,3 | 4,68E-02 | Glycine cleavage system H protein |
| argT | 2,3 | 1,48E-06 | Lysine/arginine/ornithine ABC transporter, periplasmic binding protein |
| ygiQ | 2,2 | 1,26E-04 | Protein of unknown function |
| dgcM | 2,1 | 3,51E-05 | Diguanylate cyclase |
| asnA | 2,1 | 3,43E-04 | Aspartate-ammonia ligase |
| fbp | 2,0 | 2,96E-04 | Fructose-1,6-bisphosphatase class 1 |
| yciF | 0,5 | 2,28E-05 | Putative rubrerythrin/ferritin-like metal-binding protein |
| glaH | 0,4 | 1,63E-03 | Glutarate dioxygenase |
| ybjP | 0,4 | 4,96E-02 | DUF3828 domain-containing lipoprotein |
| metF | 0,4 | 1,26E-05 | Methylenetetrahydrofolate reductase |
| phoA | 0,4 | 5,52E-05 | Alkaline phosphatase |
| lldD | 0,2 | 1,80E-06 | L-lactate dehydrogenase |
| metE | 0,1 | 1,89E-10 | Cobalamin-independent homocysteine transmethylase |
| nhaA | 0,0 | 1,10E-02 | Na^+^:H^+^ antiporter |

**Supplementary Table 3.** List of the *E*. *coli* K-12 W3110 WT proteins that were differentially accumulated in M9 glycerol EA B12 compared to M9 glycerol NH_4_Cl (fold-change > 2 or < 0,5 and p-value < 0,05). The annotated functions as indicated on STRING v11.5 are also reported.

|  | Protein | Fold-change | p-value | Predicted function |
| --- | --- | --- | --- | --- |
|  | NudK | ND | ND | GDP-mannose hydrolase |
|  | YpfG | ND | ND | Unknown |
|  | TktB | 0,69 | 4,27E-05 | Transketolase |
|  | TalA | 0,68 | 1,77E-04 | Transaldolase |
|  | MaeB | 1,00 | 8,78E-01 | Malic enzyme |
|  | HemF | ND | ND | Coproporphyrinogen III oxidase |
|  | AmiA | 1,20 | 2,63E-02 | N-acetylmuramoyl-L-alanine amidase |
|  | YpeA | 0,96 | 1,29E-01 | Acetyltransferase (GNAT) family |
|  | YfeZ | ND | ND | Unknown |
|  | YfeY | 0,87 | 1,09E-02 | Unknown |
|  | YfeX | 0,88 | 2,05E-02 | Dye-decolorizing peroxidase |

**Supplementary Table 4.** Abundance of the detected EUT1 locus ancillary proteins in M9 glycerol EA B12 compared to M9 glycerol NH_4_Cl. None of the detected EUT1 locus ancillary proteins passed the set significance thresholds (fold-change > 2 or < 0,5 and p-value < 0,05). ND: not detected.

| **Reaction** | **Description** | **Localization** | **Value** |
| --- | --- | --- | --- |
| **Experimentally determined values** | | | |
| µ | Growth rate (h^-1^) | NA | 0.37 ± 0.01 |
| q_S)Glycerol_ | Glycerol uptake from extracellular medium | Extracellular | 13.68 ± 0.15 |
| q_S)Ethanolamine_ | Ethanolamine uptake from extracellular medium | Extracellular | 7.04 ± 0.23 |
| q_P)Ethanol_ | Ethanol excretion into extracellular medium | Cytosol | 2.67 ± 0.11 |
| q_P)Acetate_ | Acetate excretion into extracellular medium | Cytosol | 1.64 ± 0.09 |
| q_P)NH4_ | Ammonium excretion into extracellular medium | Extracellular | 0.86 ± 0.21 |
| **Predicted values for ethanolamine-derived acetaldehyde metabolism** | | | |
| v_EutBC_ | EAL | BMC | 7.04 ± 0.23 |
| v_eBMC)Acetaldehyde_ | Acetaldehyde leakage from the Eut BMCs | BMC | 1.70 ± 0.11 |
| v_EutG_ | Acetaldehyde to ethanol conversion (NAD^+^-dependent) | BMC | 2.67 ± 0.11 |
| v_eBMC)Ethanol_ | Ethanol diffusion out of the BMC | BMC | 2.67 ± 0.11 |
| v_EutE_ | Acetaldehyde to acetyl-CoA conversion (NADH-dependent) | BMC | 2.67 ± 0.11 |
| v_EutD_ | Acetyl-CoA to acetyl-P conversion | BMC | 2.67 ± 0.11 |
| v_eBMC)Acetyl-P_ | Acetyl-P diffusion out of the BMC | BMC | 2.67 ± 0.11 |
| **Predicted values for glycerol metabolism** | | | |
| V_gly)biomass_ | Biomass production from glycerol | Cytosol | 11.17 ± 0.20 |
| V_gly)glycolysis_ | Acetyl-P production from glycerol | Cytosol | 2.51 ± 0.06 |
| **Predicted values for cytosolic acetyl-P metabolism** | | | |
| v_EutP, EutQ, AckA_ | Acetyl-P to acetate conversion | Cytosol | 1.64 ± 0.09 |
| v_Pta_ | Acetyl-P to acetyl-CoA (to biomass) conversion | Cytosol | 3.54 ± 0.14 |
| **Predicted values for ethanolamine-derived ammonium metabolism** | | | |
| v_eBMC)NH4_ | Ammonium diffusion out of the BMC | BMC | 7.04 ± 0.23 |
| v_NH4)biomass_ | Biomass production from ammonium | Cytosol | 6.18 ± 0.08 |

**Supplementary Table 5.** Growth parameters of *E*. *coli* K-12 W3110 WT cultivated aerobically in M9 medium containing ^12^C_2_-glycerol, ^13^C_2_-EA and cyanocobalamin. The experimentally determined substrate uptake (q_S_) and product excretion (q_P_) rates are expressed in mmol.(gDW.h)^-1^. Intracellular flux values (v) determined using the isotopic model are also expressed in mmol.(gDW.h)^-1^. DW: dry weight; NA: not appliable. Data is the average of n=3 independent replicates, errors indicate SD.
